# Preparation and Characteristics of Porous Mullite Ceramics by 3D Printing and In-Situ Synthesis

**DOI:** 10.3390/ma18050956

**Published:** 2025-02-21

**Authors:** Rina Wu, Chaochao Wang, Guodong Xu, Meiling Fan, Zhigang Huang, Tao Zeng, Xiaohong Wang

**Affiliations:** 1 School of Materials Science and Chemical Engineering, Harbin University of Science and Technology, Harbin 150080, China; 15776749213@163.com (R.W.); 15546071791@163.com (M.F.); 2 Department of Civil and Environmental Engineering, College of Engineering, Shantou University, Shantou 515063, China; jxepdi-wangchaochao@powerchina.cn (C.W.); 24zghuang@stu.edu.cn (Z.H.); taozeng@hrbust.edu.cn (T.Z.); xiaohongwang@stu.edu.cn (X.W.); 3 Powerchina Jangxi Electric Power Design Institute Co., Ltd., Nanchang 330096, China; 4 Special Ceramics Advanced Manufacturing Engineering Technology Research Center of Guangdong Provincial University, Shantou 515063, China

**Keywords:** mullite ceramic, 3D printing, rheological property, flexural behaviors, pore structure

## Abstract

In-situ porous mullite ceramics with varying pore size and porosity were fabricated using 3D printing. The pore size was controlled by adjusting the size of polymethyl methacrylate (PMMA) microspheres. The effect of sintering temperature on phase evolution was also examined. Additionally, the impact of PMMA microsphere size and content on the rheological properties of the printing inks was investigated. The results indicated that alumina and microsilica fully transformed into mullite at 1550 °C. The influence of PMMA microsphere content and size on the porosity, mechanical properties, and thermal conductivity of 3D-printed porous mullite ceramics was also studied. The 3D-printed porous mullite ceramic prepared with 15 μm PMMA microspheres exhibited a porosity of 44.38%, a flexural strength of 58.53 MPa, and a thermal conductivity of 2.21 W/(m·K). This printing strategy offers a simple and effective method for fabricating porous mullite ceramics.

## 1. Introduction

Porous mullite ceramics (3Al_2_O_3_⋅2SiO_2_) have attracted attention in many advanced industrial applications [1,2]. The unique properties of porous mullite ceramics enable them to maintain good physical and chemical stability at elevated temperatures. In addition, they exhibit excellent resistance to thermal shock and low thermal conductivity [3,4]. These properties render them ideal for high-temperature industrial applications, such as heat exchangers and combustion chamber linings. Moreover, porous mullite ceramics also excel in the filtration field and are often used as highly efficient filtration media in gas or liquid purification processes, effectively removing fine particles and hazardous substances and making a significant contribution to environmental protection [5,6,7]. In addition, due to the material’s good biocompatibility and corrosion resistance, it is also being explored for applications in more emerging fields, including but not limited to bone replacement materials or catalyst carriers in the medical industry, showing a broad application prospect [8,9]. Although many conventional techniques, such as gel casting, pore blowing agents, sacrificial templates, freeze casting, and direct foaming [10,11,12,13,14], have been used to produce porous mullite ceramics, these methods are often limited by molds and processing tools, making it difficult to achieve complex and customized geometries.

Recently, 3D printing has emerged as a technology based on point-by-point, line-by-line, or layer-by-layer fabrication without molds, offering remarkable flexibility for manufacturing ceramics with customized shapes and intricate internal structures [15,16,17,18]. He et al. [19] used digital light processing (DLP) to fabricate porous mullite ceramic from a preceramic polymer precursor containing alumina particles. Schmidt et al. [20] employed stereolithography (SL) to prepare mullite complex structures using a silicone preceramic polymer with alumina particles. Gorjan et al. [21] explored the in-situ fabrication of honeycomb mullite structures using fused deposition modeling (FDM) 3D printing technique. Chen et al. [22] fabricated porous mullite ceramics using selective laser sintering (SLS). Li et al. [23] produced lightweight mullite ceramics with 85% porosity and 2.08 MPa compressive strength using fly ash hollow spheres and polyamide-12 powder via SLS. Ma et al. [24] combined SLA 3D printing with a reaction sintering method to in-situ fabricate porous honeycomb mullite ceramics. Compared to other additive manufacturing methods, direct ink writing (DIW) offers distinct advantages for manufacturing mullite ceramic structures due to its simplicity, cost-effectiveness, wide material adaptability, and high degree of customization, allowing for both uniformly distributed micropores and irregularly arranged macro-pores to meet specific needs in various engineering fields.

Many studies have been conducted on the fabrication of porous mullite using DIW technology. Man et al. [25] developed lightweight mullite boards via DIW, employing α-Al_2_O_3_ and kaolin clay as raw materials. Hossain et al. [26] fabricated mullite foam using waste rice husk ash through the DIW process. Yang et al. [27] utilized fly ash hollow spheres to produce a high-strength mullite-based lattice by DIW, which achieved a porosity of 84.3%, a compressive strength of 2.74 MPa, and a thermal conductivity of 0.191 W/(m·K). Li et al. [28] prepared complex ceramics of mullite lightweight refractory materials, exhibiting a high compressive strength and a low thermal conductivity via DIW. However, the aforementioned studies primarily controlled porosity by adjusting macrostructures or employing single-sized hollow sphere particles as raw materials. Research on DIW strategies for precise control of micropore characteristics, such as porosity and pore size, in porous mullite ceramics to meet the high-precision requirements of various applications remain limited.

The present study focuses on the preparation of the porous mullite ceramics with tailored porosity using DIW combined with reaction sintering. To control the pore size and shape of the porous mullite ceramics, polymethyl methacrylate (PMMA) microspheres were selected as pore-forming agents. The effect of PMMA microsphere content and size on the porosity, mechanical properties, and thermal conductivity of the porous mullite ceramics was investigated.

## 2. Materials and Methods

### 2.1. Raw Materials

**Figure 1:**
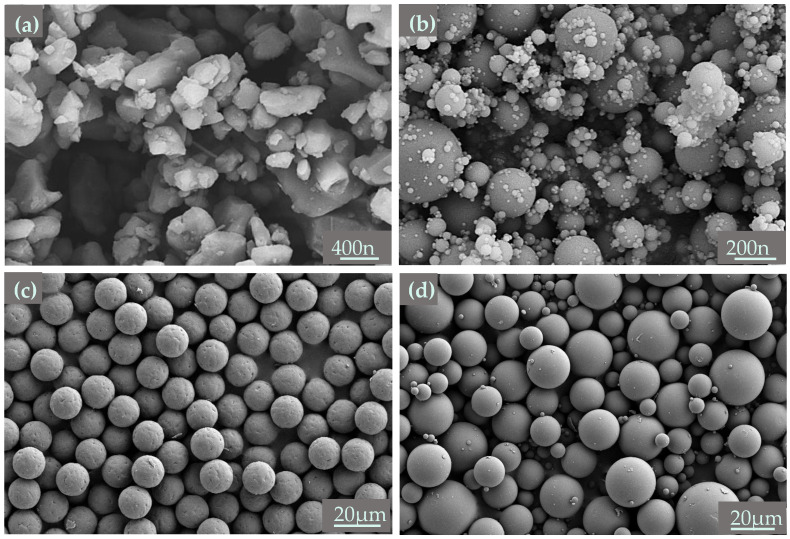
SEM morphologies of (**a**) Al_2_O_3_ powders, (**b**) 3 mol % Y20: stabilized microsilicon powders, (**c**) PMMA microspheres with 15 μm particle size, and (**d**) PMMA microspheres with 30 μm particle size.

The raw materials utilized for preparing mullite ceramic were commercial α-Al_2_O_3_ ceramic powders (D_50_ = 0.5 μm, purity > 99.9%, Fujian Choloon Import and Export Trade Co., Ltd., Quanzhou, China) and 3 mol % Y20: stabilized microsilicon powders (D_50_ = 0.1–0.3 μm, purity > 91.3%, Yuanxiang Water Purification Material Co., Ltd., Gongyi, China). PMMA microspheres (Mingyuxing Plastic Raw Materials Co., Ltd., Dongguan, China) were used as pore-forming agents with average diameter of 5–30 μm. Figure 1 shows the SEM morphologies of Al_2_O_3_ powders, 3 mol % Y20: stabilized microsilicon powders, and PMMA microspheres with 15 μm and 30 μm. Hydroxypropyl methyl cellulose (HPMC, Yien Chemical Technology Co., Ltd., Shanghai, China) was used as a binder, while trisodium citrate dihydrate (Fuchen Chemical Reagent Co., Ltd., Tianjin, China) was selected as the dispersant.

### 2.2. Preparation of Porous Mullite Ceramics

**Figure 2:**
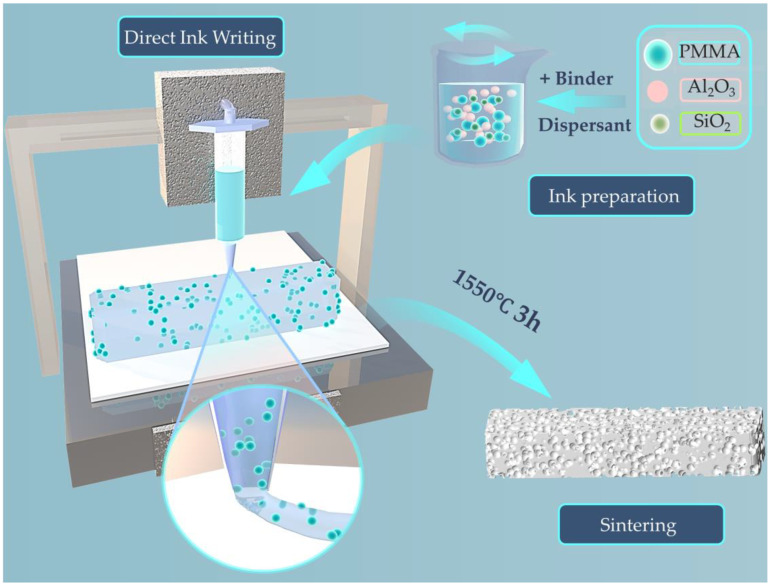
Schematic illustration of fabrication process of porous mullite ceramics by DIW 3D printing technology.

The process flow for preparing porous mullite ceramics using direct ink writing (DIW) is illustrated in Figure 2. Firstly, alumina and microsilica powders were mixed at a molar ratio of 3:2.5. Next, 10–70 vol.% PMMA microspheres were added into the mixed powders. The mixtures were added into the deionized water with 0–1.5 wt.% HPMC and 1 wt.% trisodium citrate dihydrate. The amount of deionized water added should ensure a solid phase (including alumina, microsilica powders, and PMMA microspheres) content of 50 vol.%. Then, the obtained pastes were subjected to a centrifuge (ZYMC-180HV, Zhongyi Technology Co., Ltd., Shenzhen, China) and initially centrifuged at 1800 rpm for 60 s to achieve preliminary mixing. Subsequently, the paste undergoes high-speed centrifugation at 3600 rpm for 45 s to ensure complete dispersion of its components. The green bodies of porous mullite ceramics were printed using a DIW extrusion device (Adventure 3D-LB-Printer-0005, Adventure Technology Co., Ltd., Shenzhen, China) with nozzles having a diameter of 410 μm. Based on investigations into the rheological behavior of mullite ceramic inks, the extrusion pressure is set to 50 psi, and the printing speed is maintained between 10 and 18 mm/s for optimal print quality and structural integrity. The printed green body was transferred to a constant temperature and humidity drying chamber. And drying program temperature was configured with a temperature setting of 30 °C and the relative humidity was gradually decreased from 90% to 60%. Finally, the 3D printed green bodies were placed into a muffle furnace for degreasing and sintering. Degreasing was conducted at 550 °C for 1 h, with a heating rate of 3 °C/min to burn out the pore-forming agents and organic materials. Subsequently, the temperature was raised to the target temperature (1450–1550 °C) at the same heating rate, with a holding time of 3 h.

### 2.3. Characterization

Rheological behaviors were measured at 25 °C by a- Hybrid Rheometer-10 (TA Instruments, New Castle, DE, USA), with a gap of 1000 μm between the parallel plates (diameter 40 mm). By adjusting the shear rate from 0.01 to 100 s^−1^, the viscosity values of each mullite ceramic inks were measured. In the oscillatory mode, we systematically evaluated the storage modulus and loss modulus as functions of shear strain. The shear strain was set to range from 0.1% to 200%. The phase composition of the 3D printed porous mullite ceramics sintered at 1450–1550 °C was characterized using X-ray diffraction (XRD; Rigaku D/Max-2400, Tokyo, Japan). The morphological characteristics of the sintered porous mullite ceramics were investigated by scanning electron microscopy (SEM, Gemini 300, Zeiss, Oberkochen, Germany). Apparent density and total porosity were evaluated by Archimedes method.

**Figure 3:**
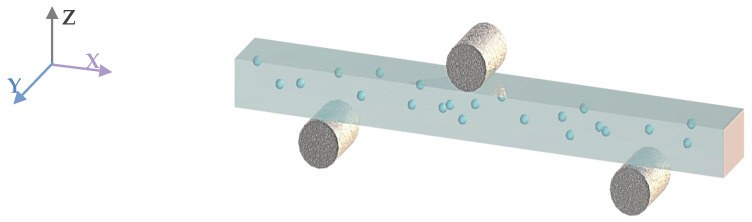
Configuration of a 3D printed porous mullite ceramic beam in three-point bending.

The flexural properties were evaluated using a universal testing machine (MTS-E45, Shenzhen, China), as configured in Figure 3. The crosshead speed was maintained at 0.5 mm/min. And the dimensions of 3D printed porous mullite ceramics were 36 mm × 4 mm × 3 mm. The span of the supports was 30 mm. At least five specimens were measured for porous mullite ceramic to obtain the average flexural strength.

(1) $$\sigma_{f}=\frac{3FL}{2bH^{2}}$$

where F is the failure load, and L, b, and H refer span length, the width, and height of the beam, respectively.

A thermal conductivity meter (DXF500, TA Instruments, New Castle, DE, USA) was used to assess the room temperature thermal conductivity of 3D printed porous mullite ceramics. The thermal conductivity was calculated as:

(2) $$K=\alpha \rho C_{p}$$

where K is the thermal conductivity, ρ is the bulk density, $C_{p}$ denotes specific heat, and α refers to the thermal diffusion coefficient.

### 2.4. Numerical Simulations

**Figure 4:**
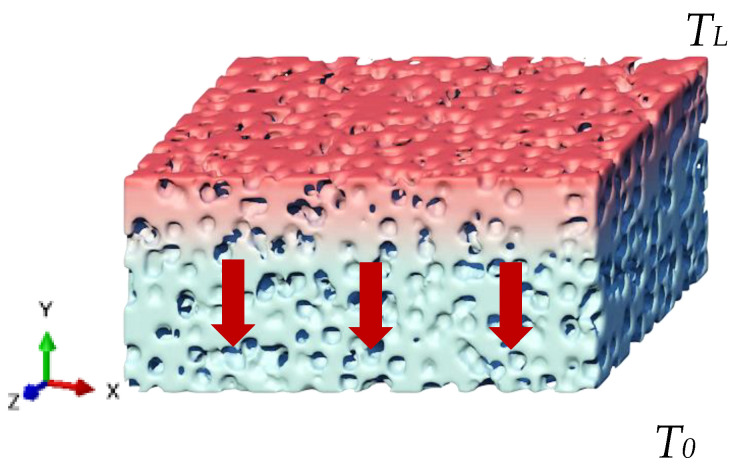
Heat conduction diagram of a porous mullite ceramic bulk.

The ABAQUS (CAE 2021) is employed to investigate the thermal conductivity of 3D printed mullite porous ceramics, as shown in Figure 4. The 3D printed porous mullite ceramic model has dimensions of 300 μm × 300 μm × 300 μm and the diameter of the pores is 15 μm. The thermal conductivity and density of the mullite are 5.73 W/(m⋅K) and 2800 kg/m^3^, respectively. The thermal conductivity of the pores is set to 0.023 W/(m⋅K) and the density is set to 1.29 kg/m^3^ [29]. The porous ceramics are considered as isotropic materials. The thermal boundary conditions are set as *T*(0, 0, 0) = *T*_0_ and *T*(0, *L_y_*, 0) = *T_L_*. According to the research by Alexander M. Thiele et al. [30], the average heat flux density (${\bar{q}}_{y}$) and effective thermal conductivity ($k_{\mathrm{eff}}$) in the *y*-direction can be obtained as follows:

(3) $${\bar{q}}_{y}=\frac{1}{A_{c}}\iint q_{y}\left(x,z\right)dxdz$$

(4) $$k_{eff}=-\frac{{\bar{q}}_{y}L}{T_{L}-T_{0}}$$

where $A_{c}$ represents the cross-sectional area of heat flow perpendicular to the *y*-axis and *L* is the unit cell length.

## 3. Results and Discussion

### 3.1. Rheological Properties of Mullite Ceramic Inks

**Figure 5:**
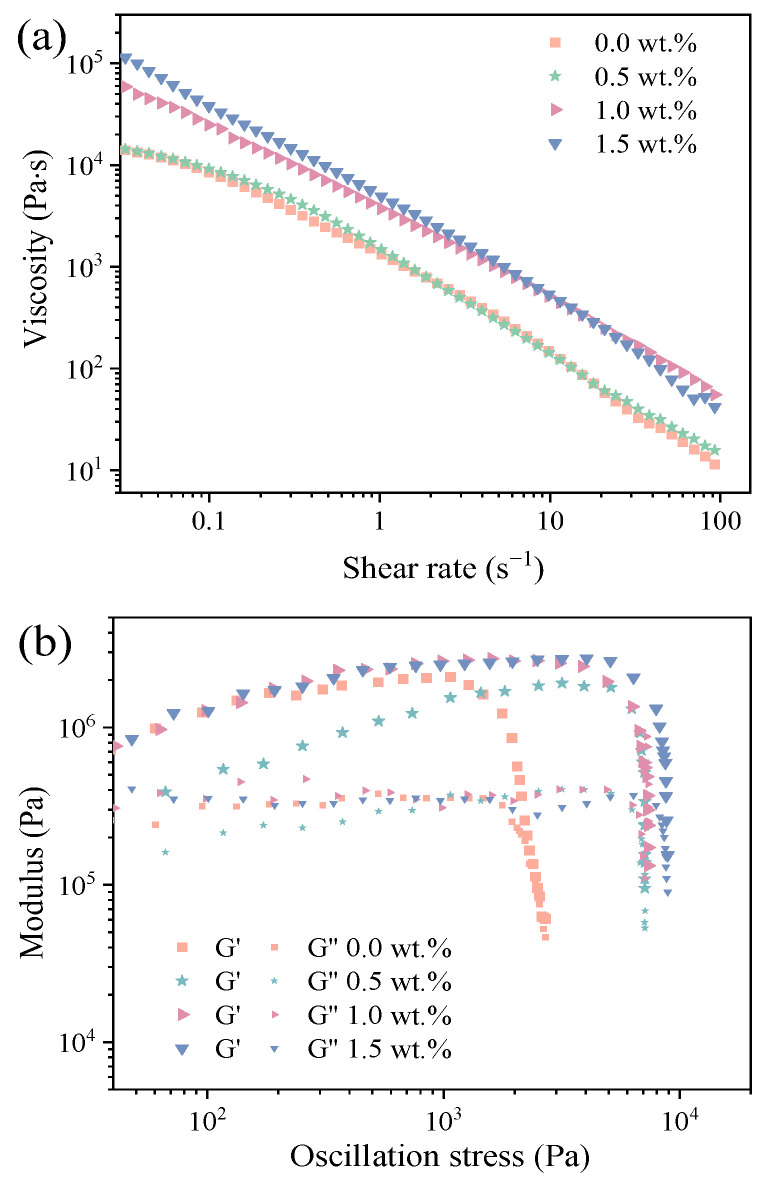
Rheological properties of mullite ceramics inks with varying HPMC contents: (**a**) viscosity, and (**b**) storage (G′) and loss (G″) modulus.

Figure 5 presents the rheological behaviors of mullite ceramic inks with varying HPMC contents at a shear rate of 0.01–100 s^−1^. As demonstrated in Figure 5a, the viscosity of ceramic inks decreased significantly with an increasing shear rate, indicating a typical shear-thinning behavior. As the HPMC content increased from 0.5 wt.% to 1.5 wt.%, zero-shear viscosity (η0) values of the ceramic inks rose from 9210.86 to 107,645 Pa·s. A sufficiently high storage modulus plateau was observed in all curves of ceramic inks from Figure 5b. All the inks exhibited pronounced yield-pseudoplastic behavior. Furthermore, the yield stress increased with the increase in HPMC content and the slurry with a binder content of 1.0 wt.% exhibited the best printability and formability.

**Figure 6:**
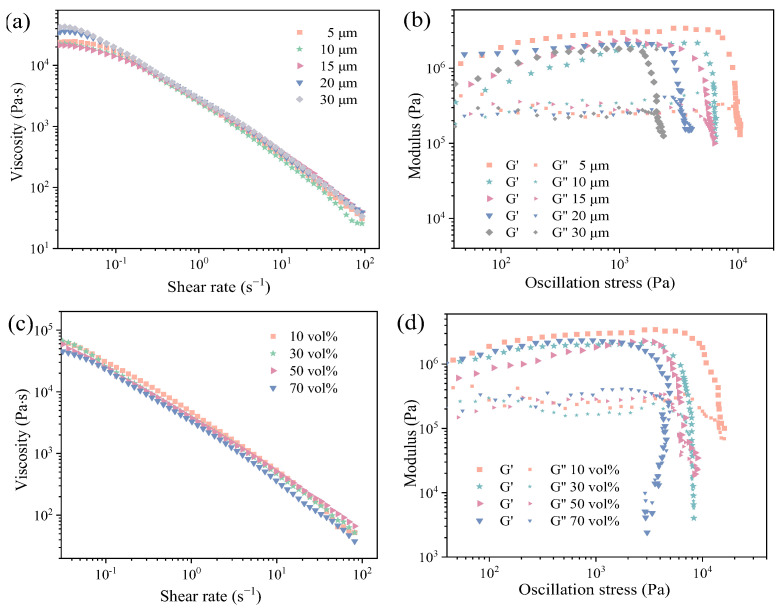
Rheological properties of mullite ceramics inks with varying PMMA microsphere size: (**a**) viscosity and (**b**) storage (G′) and loss (G″) modulus, and mullite ceramics inks with varying PMMA contents: (**c**) viscosity, and (**d**) storage (G′) and loss (G″) modulus.

Figure 6 presents the rheological behavior of ceramic inks with varying PMMA microsphere sizes. As noted in Figure 6a, as the diameter of PMMA increases from 5 μm to 30 μm, the value of zero-shear viscosity (η0) increases. From Figure 6b, it is clearly evident that as the diameter of PMMA increases, the storage modulus plateau values slightly decrease under low shear stress. Concurrently, the yield stress of the slurry exhibits a decreasing trend. Figure 6c,d presents the rheological behaviors of ceramic inks with varying PMMA contents. When the content of PMMA increases from 10 vol.% to 70 vol.%, the viscosity of the slurry decreases from 64,043.3 Pa·s to 45,189.4 Pa·s. This is due to the smooth surface of the PMMA microspheres, which reduces the friction between the particles [31]. Additionally, the addition of PMMA microspheres improves the sphericity of the solid phase, significantly reducing the movement resistance [32] particles and leading to a decrease in slurry viscosity as the PMMA content increases. In Figure 6d, it is evident that at low shear stress, ceramic inks with varying PMMA microspheres display similar high storage modulus plateau values. This characteristic is favorable for maintaining the geometrical shape and structural stability of the printed specimens. As the PMMA content increases, the yield stress of mullite ceramic slurries decreases.

### 3.2. Phase Composition Analysis

**Figure 7:**
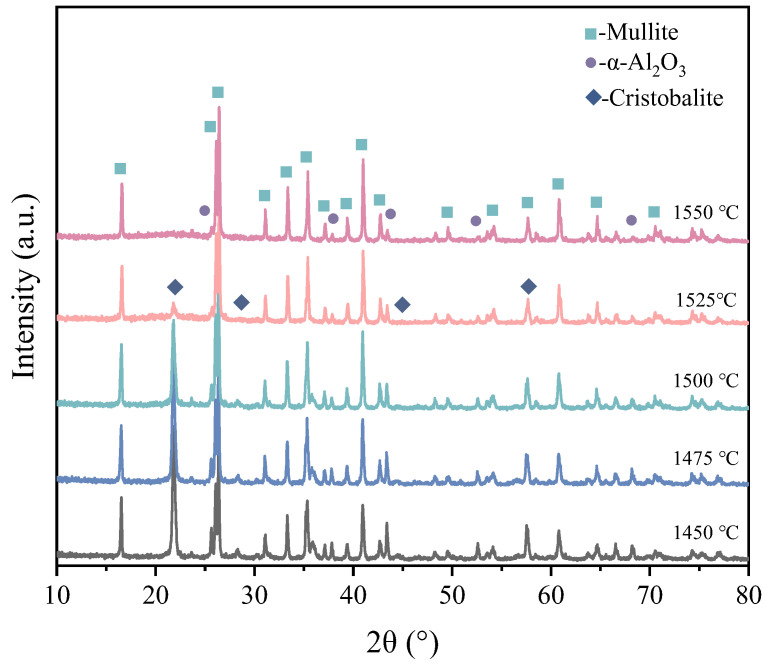
XRD patterns of 3D printed porous mullite ceramics sintered at 1450 °C, 1475 °C, 1500 °C, 1525 °C, and 1550 °C for 3 h.

Figure 7 illustrates the XRD patterns of 3D printed porous mullite ceramics sintered at 1450–1550 °C with each phase content determined by the Rietveld analysis. The sample sintered at 1450 °C for 3 h primarily composed of alumina and mullite, with mullite constituting 63.1 wt.% and cristobalite 19.8 wt.%. For the 3D printed porous mullite ceramics sintered at 1500 °C, the cristobalite peak diminished, while mullite content increased to 75.0 wt.%. At 1550 °C, the cristobalite peak disappeared and the phase composition was dominated by alumina and mullite. At this temperature, the mullitization reaction was observed to be essentially completed, with an α-alumina content of 6.8 wt.% and a mullite content of 93.2 wt.%. Based on the XRD results, the optimal sintering temperature is determined to be 1550 °C.

### 3.3. Effect of PMMA Size

**Figure 8:**
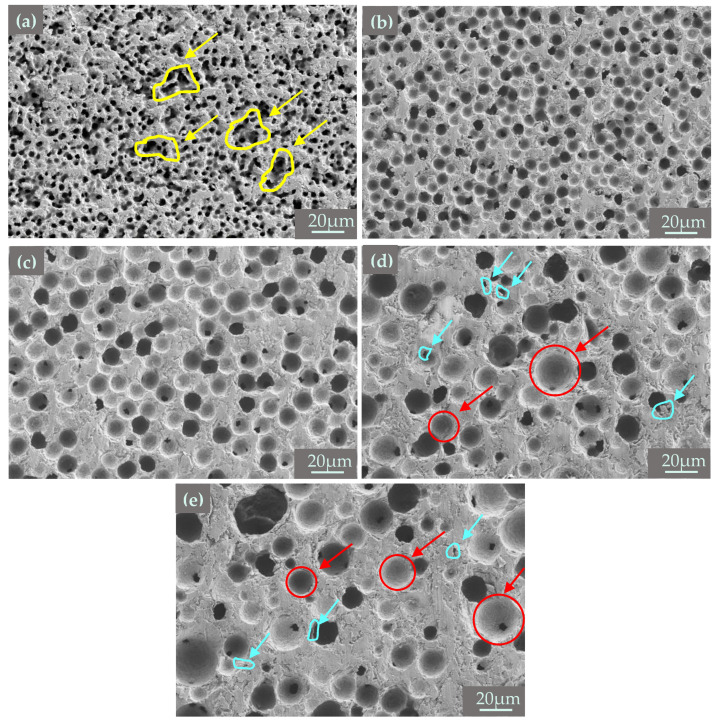
SEM micrographs of 3D printed porous mullite ceramics with varying PMMA microsphere sizes: (**a**) 5 μm, (**b**) 10 μm, (**c**) 15 μm, (**d**) 20 μm, and (**e**) 30 μm.

**Figure 9:**
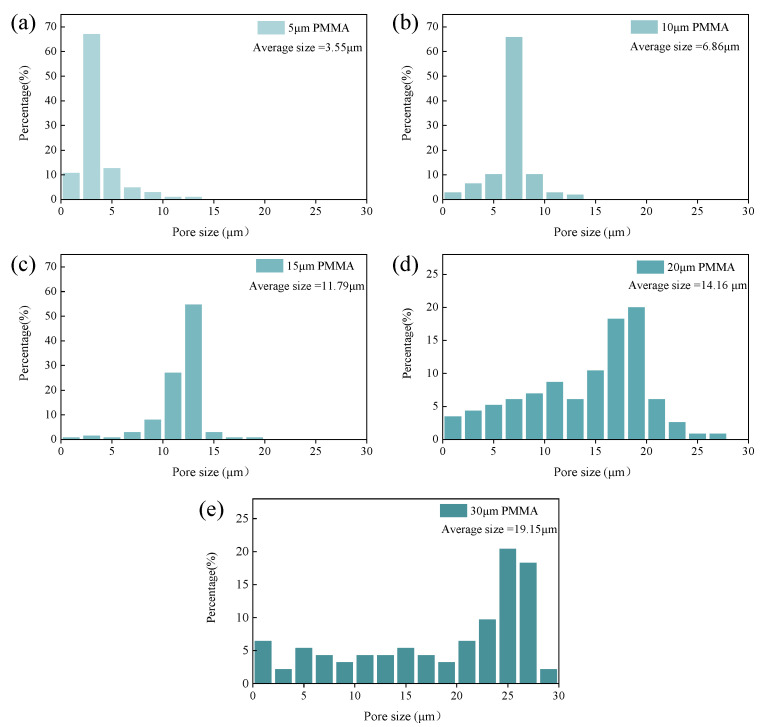
Pore size distribution of 3D printed porous mullite ceramics with varying PMMA microsphere sizes: (**a**) 5 μm, (**b**) 10 μm, (**c**) 15 μm, (**d**) 20 μm, and (**e**) 30 μm.

Figure 8 shows the microstructure of 3D printed porous mullite ceramics with varying PMMA microsphere sizes. And the pore distribution, evaluated using NanoMeasurer software 1.2, is shown in Figure 9. The PMMA content in all samples is 50 vol.%. The average pore size in sintered samples prepared with 5 μm PMMA microspheres is 3.55 μm (Figure 9a) and the pore distribution is uneven with aggregation (shown by yellow arrows). As the particle size of PMMA microspheres increases to 15 μm, the pores in the porous mullite ceramics prepared show a better ordering. From SEM images of mullite ceramics with 20 μm and 30 μm PMMA microspheres, it can be observed that larger, circular pores are formed after the removal of PMMA (show with red arrows), along with irregular pores (show with blue arrows). This phenomenon is due to the fact that the removal of PMMA leads to the formation of a pore structure in mullite ceramic, which inhibits densification, and the inhibition is more pronounced in porous samples with larger pores [33].

**Figure 10:**
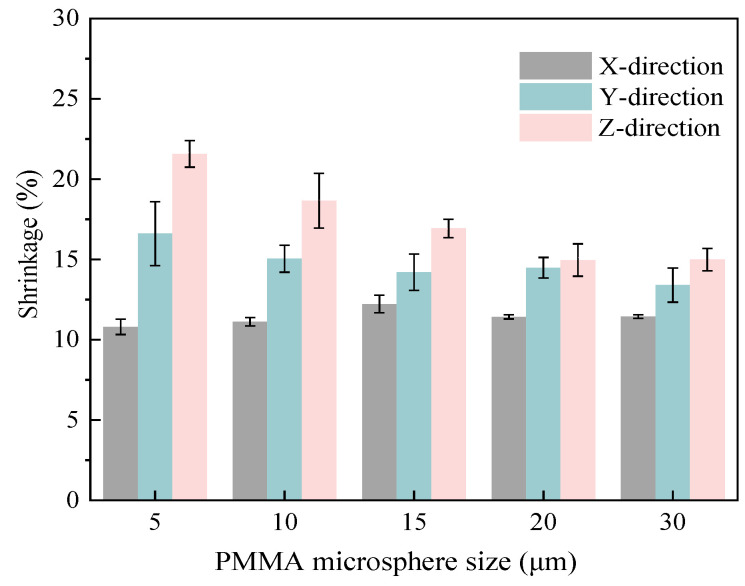
Shrinkage of mullite porous ceramics with varying PMMA microsphere sizes.

Figure 10 shows variation of shrinkage in all directions for mullite porous ceramics with varying PMMA microspheres sizes. As the PMMA microsphere particle size increases, the shrinkage of mullite porous ceramics in the Y-direction and Z-direction first decreases and then tends to level off, while the shrinkage in the X-direction changes less. When the particle size of PMMA microspheres was increased from 5 μm to 30 μm, the X-direction shrinkage increased from 10.80% to 11.21%, the Y-direction shrinkage decreased from 16.61% to 13.40%, and the Z-direction shrinkage decreased from 21.57% to 14.99%.

**Figure 11:**
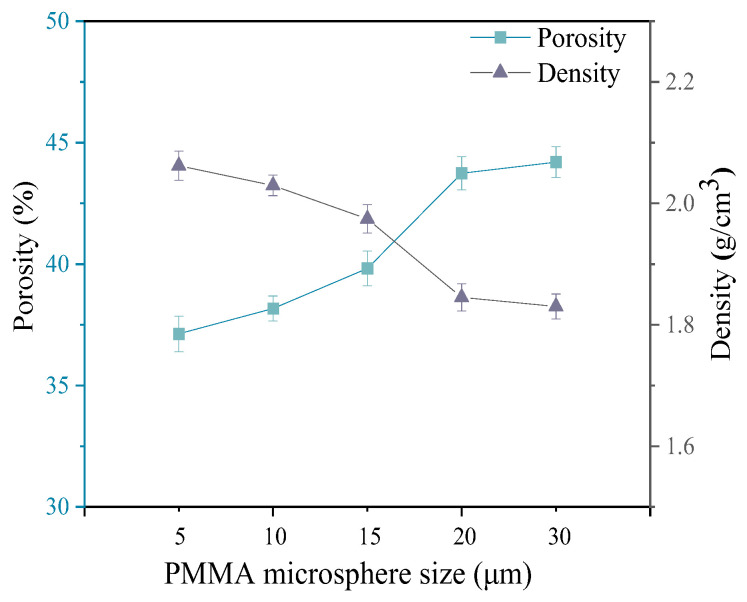
Porosity and density for 3D printed porous mullite ceramics with varying PMMA microsphere sizes.

The density and porosity of 3D printed porous mullite ceramics with varying PMMA microsphere sizes are presented in Figure 11. As the PMMA microsphere size increases from 5 μm to 30 μm, the porosity of the 3D printed porous mullite ceramics increases from 37.13% to 44.20%, while the density decreases from 2.06 to 1.83 g/cm^3^.

**Figure 12:**
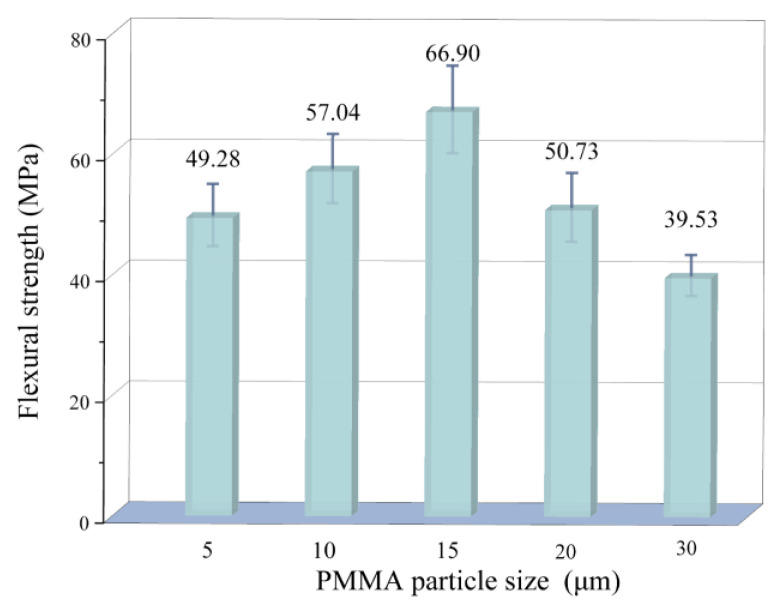
Flexural strength of 3D printed porous mullite ceramics with varying PMMA microsphere sizes.

Figure 12 illustrates the trend of 3D printed porous mullite ceramic flexural strength with the PMMA microsphere size. The flexural strength of 3D printed mullite porous ceramics firstly increases and then decreases with the increase of PMMA microsphere size. In fact, both the porosity and pore distribution modulate the flexural properties of 3D printed porous ceramics [34]. For the 3D printed porous mullite ceramics with a PMMA microsphere particle size of 5 μm, although its overall porosity is the lowest, some areas still contain a large number or larger pores (Figure 8a), leading to poor flexural strength. The flexural strength reaches a maximum value of 66.9 MPa when the PMMA microsphere size is 15 μm. The reason is that the pores generated in the sample containing 15 μm PMMA microspheres are more uniformly distributed compared to those in the sample with 5 μm PMMA microspheres. When the PMMA microsphere size is 30 μm, the flexural strength is 39.53 MPa. As the PMMA microsphere size continues to increase, the oversized pores inhibit densification process, significantly reducing flexural strength.

**Figure 13:**
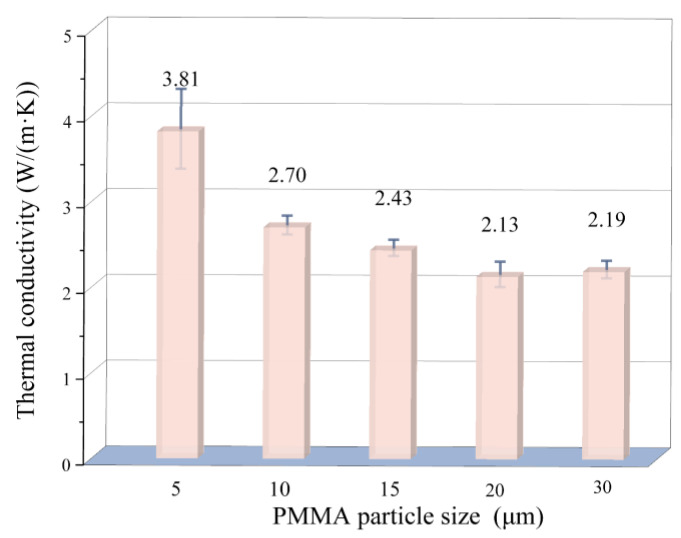
Room temperature thermal conductivity of 3D printed porous mullite ceramics with varying PMMA microsphere sizes.

Figure 13 shows the effect of PMMA microsphere size on the thermal conductivity of porous mullite ceramics. When the average diameter of PMMA microspheres increases from 5 μm to 20 μm, the thermal conductivity of the porous mullite ceramics decreases from 3.81 W/(m·K) to 2.13 W/(m·K). When the PMMA size further increases to 30 μm, the thermal conductivity slightly increases. This behavior can be attributed to the complex interplay between porosity and pore size. On one hand, the increase in pore size inhibits the densification process of the porous mullite pore walls, thereby increasing overall porosity. This increase in porosity leads to a reduction in the effective thermal conductivity path and a blockage of heat transfer, which significantly reduces the thermal conductivity. On the other hand, although the porosity of the mullite porous ceramics with 20 μm PMMA microspheres is 43.74% and that with 30 μm PMMA microspheres is 44.2% (a minor change). For similar porosity levels, larger pore sizes and fewer pores facilitate enhanced heat transfer between the solid phases, thereby increasing thermal conductivity [35,36].

### 3.4. Effect of PMMA Content

**Figure 14:**
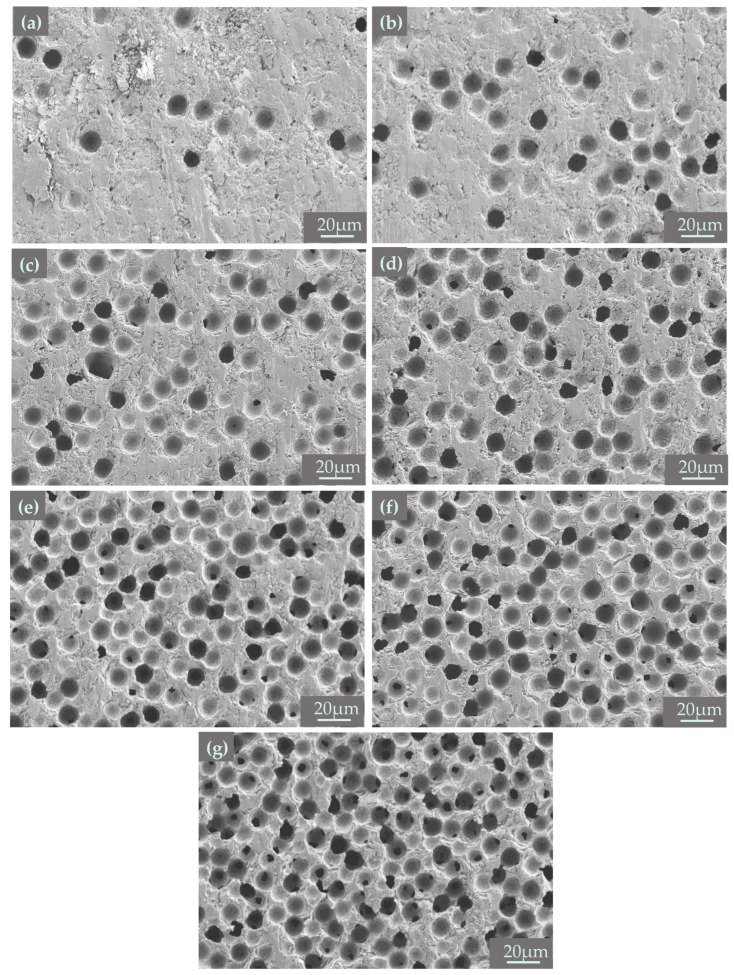
Microstructures of 3D printed porous mullite ceramics with varying PMMA content: (**a**) 10 vol.%, (**b**) 20 vol.%, (**c**) 30 vol.%, (**d**) 40 vol.%, (**e**) 50 vol.%, (**f**) 60 vol.%, and (**g**) 70 vol.%.

**Figure 15:**
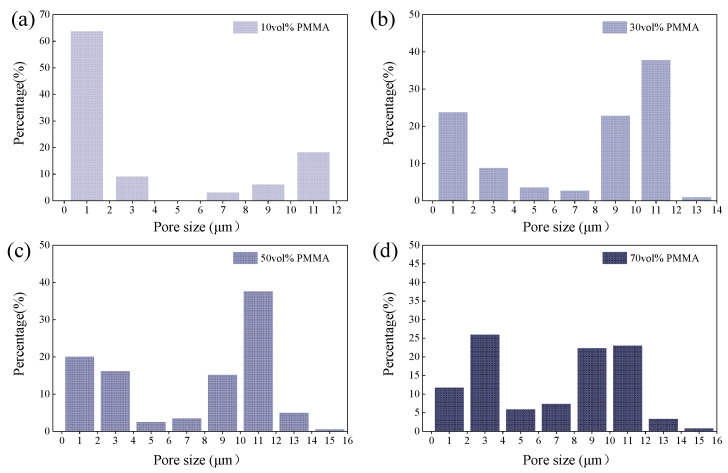
Pore size distribution of 3D printed porous mullite ceramics with varying PMMA content: (**a**) 10 vol.%, (**b**) 30 vol.%, (**c**) 50 vol.%, and (**d**) 70 vol.%.

To investigate the effect of PMMA content on the microstructures of mullite porous ceramics, the PMMA microspheres with an average diameter of 15 μm were used as the pore-forming agent, and samples with PMMA microspheres content (10, 20, 30, 40, 50, 60, 70 vol.%) were printed. Figure 14 demonstrates the microstructures of 3D printed mullite porous ceramics with varying PMMA content. And the pore distribution of different PMMA microspheres content (10, 30, 50, 70 vol.%) is shown in Figure 15. It can be observed that the pores formed by PMMA retain the spherical shape during the sintering process. In the porous mullite ceramics with 10 vol.% PMMA, most of the pores are isolated (as shown in Figure 14a). The pores with sizes ranging from 0.5 to 1.5 μm, formed by particle accumulation, account for a larger proportion (as shown in Figure 15a). As PMMA content increases, the number of pores significantly increases and its distribution becomes more uniform (as shown in Figure 15d).

**Figure 16:**
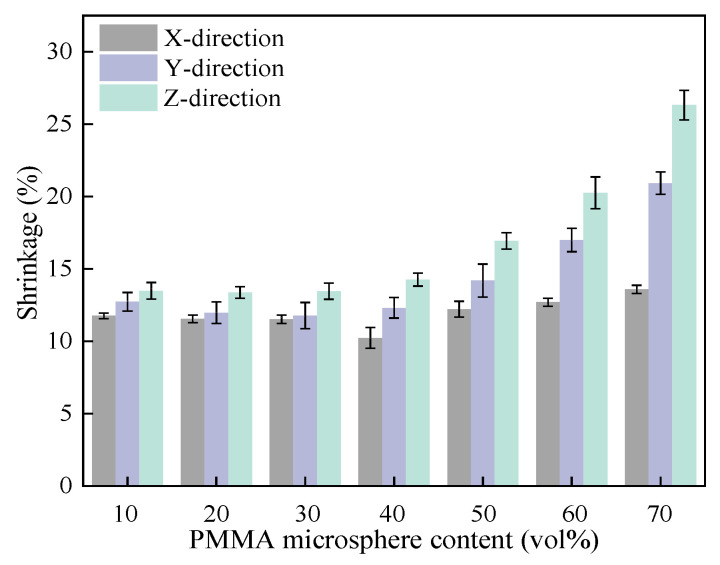
Shrinkage of mullite porous ceramics with varying PMMA microsphere contents.

Figure 16 shows variation in shrinkage of 3D printed mullite porous ceramics with varying PMMA microsphere contents. It can be observed the shrinkage of 3D printed mullite porous ceramics in all three directions shows a gradual increase with increasing PMMA microsphere content. The shrinkage of mullite porous ceramics fluctuated between 11% and 15% in three directions when the PMMA microsphere content was below 30 vol.%. The densification of the ceramic matrix is the primary cause of this shrinkage. As the content of PMMA microspheres is increased from 40 vol.% to 70 vol.%, the shrinkage of 3D printed in all directions becomes more pronounced. Shrinkage increases progressively in Y-direction and Z-direction, whereas changes in the X-direction remain relatively insignificant.

**Figure 17:**
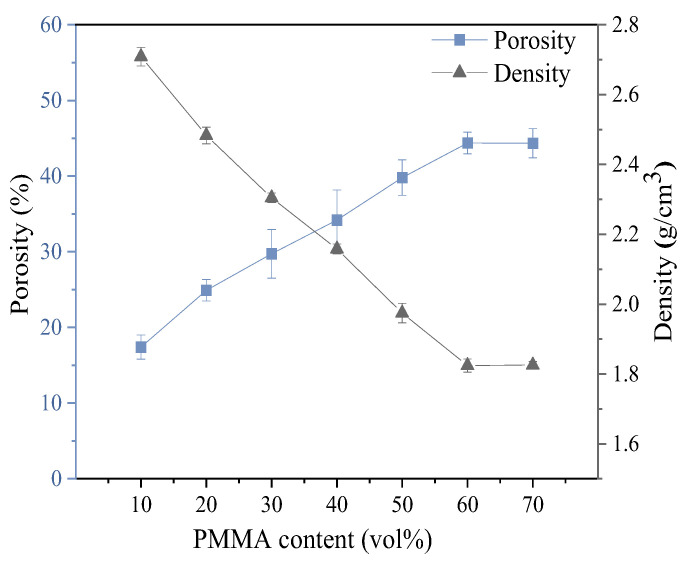
Porosity and density for 3D printed porous mullite ceramics with varying PMMA contents.

The density and porosity of porous mullite ceramics with varying PMMA content are shown in Figure 17. The results indicate that the porosity of the porous mullite ceramics first increases and then gradually stabilizes, while the density exhibits an opposite trend. When the addition of PMMA microspheres increases from 10 vol.% to 60 vol.%, the porosity increases from 17.41% to 44.48% and the density decreases from 2.70 g/cm^3^ to 1.82 g/cm^3^.

**Figure 18:**
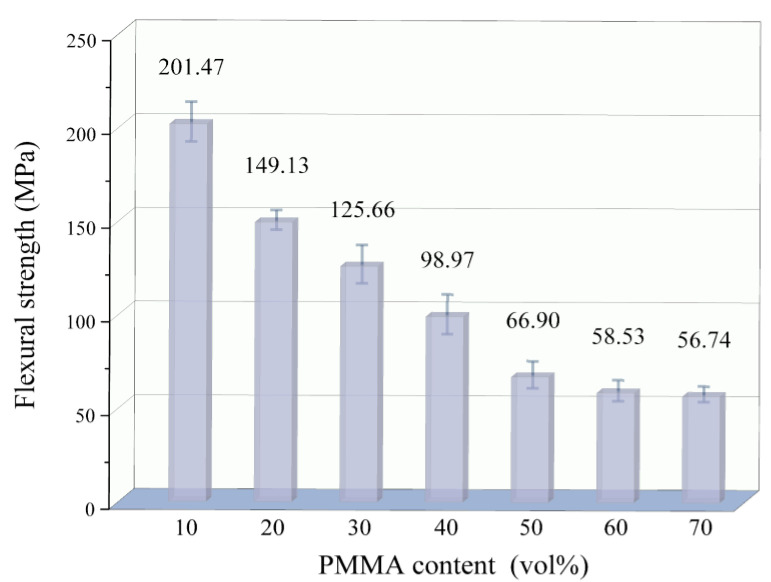
Flexural strength of 3D printed porous mullite ceramics with varying PMMA contents.

Figure 18 illustrates the trend of 3D printed porous mullite ceramic flexural strength with PMMA content. It can be observed from the figure that as the amount of PMMA microspheres increases, the flexural strength of the porous mullite ceramics initially decreases and then stabilizes. When the PMMA content increases from 10 vol.% to 60 vol.%, the flexural strength of the porous ceramics decreases from 201.47 MPa to 58.53 MPa. Further increasing the PMMA content, the flexural strength of 3D printed porous ceramic remains essentially unchanged.

**Figure 19:**
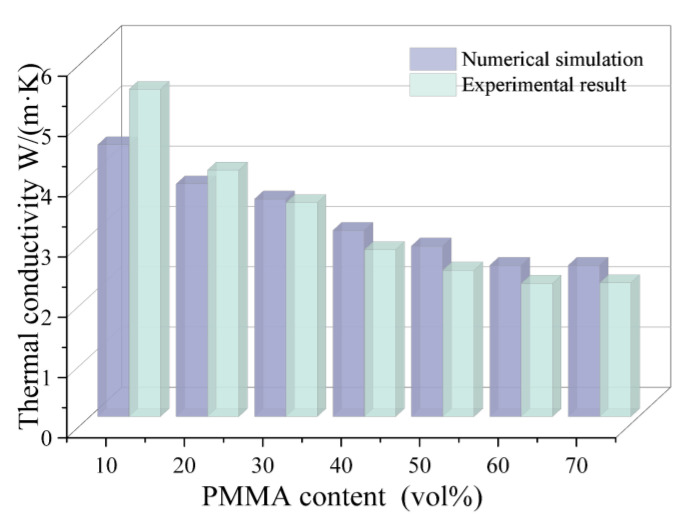
Comparison of numerical simulation and experimental results of thermal conductivity of 3D printed porous mullite ceramics with varying PMMA contents.

Figure 19 shows the effect of PMMA content on the thermal conductivity of 3D printed porous mullite ceramics. And the calculated temperature field is presented in Figure 15. It can be observed that as the PMMA content increases, the thermal conductivity of the 3D printed porous mullite ceramics gradually decreases. When the PMMA content increases from 10 vol.% to 70 vol.%, the thermal conductivity of the porous ceramics decreases from 5.43 W/(m·K) to 2.22 W/(m·K). The numerical simulation results are in good agreement with the experimental values.

The strategy facilitates the production of reliable, tailor-made porous mullite ceramics, effectively adjusting key performance parameters such as porosity, thermal conductivity, and mechanical strength. This enables porous mullite ceramics to meet specific application requirements and provide high-performance solutions in a wide range of fields. In future work, we will expand the scope of the study to include a wider variety of particle sizes, providing insight into the specific mechanisms of action and their impact on the realization of functionality in each specification. We will focus on the potential for surface discoloration and aesthetic degradation of ceramic materials when exposed to chemicals or high temperatures over long periods. These factors are particularly significant in applications, where changes in color or surface appearance can compromise the material’s functionality and aesthetic value [37]. By employing accelerated aging test methods to systematically analyze trends in mechanical strength, thermal stability, and aesthetic qualities under simulated extreme conditions such as UV irradiation, chemical attack, and thermal cycling, we aim to accurately assess the real-world performance of a wide range of ceramic products under harsh operating conditions. In addition, considering the important role of surface treatment and coating technologies in enhancing the resistance of ceramics to environmental erosion and prolonging their service life, we will strengthen our focus on this area in order to develop new ceramic materials that are more durable, have superior performance, and maintain a good appearance.

## 4. Conclusions

The present study successfully utilized 3D printing technology to fabricate in situ mullite porous mullite (3Al_2_O_3_⋅2SiO_2_) ceramics. PMMA microspheres with different sizes were used to form customized pore structures. The microstructures and properties of the samples could be flexibly controlled. After sintering the 3D-printed green body at 1550 °C for 3 h, the mullite reaction is essentially complete, resulting in an alumina content of 6.8 wt.% and a mullite content of 93.2 wt.%. As the PMMA microsphere size increases from 5 μm to 30 μm, the porosity of the porous mullite ceramics increases from 37.13% to 44.20% and the thermal conductivity decreases from 3.81 W/(m·K) to 2.19 W/(m·K). The strength initially increases and then decreases. The porous mullite ceramic with 15 μm PMMA microspheres exhibits the highest flexural strength of 66.9 MPa. Furthermore, as the PMMA content increases from 10 vol.% to 70 vol.%, the porosity of the porous mullite ceramic increases from 17.41% to 44.48% and the flexural strength decreases from 201.47 MPa to 56.74 MPa. The thermal conductivity also decreases from 5.43 W/(m·K) to 2.22 W/(m·K). The strategy facilitates the production of reliable, tailor-made porous mullite ceramics, effectively adjusting key performance parameters such as porosity, thermal conductivity, and mechanical strength. This enables porous mullite ceramics to meet specific application requirements and provide high-performance solutions in a wide range of fields. Nevertheless, there are still limitations in the present work. The actual service life of these new materials and their stability under long-term operating conditions have yet to be accumulated and verified. Future research will focus on expanding the scope of the experiments to include longer time spans and to consider more diverse application environments.

## Data Availability

The original contributions presented in this study are included in the article material. Further inquiries can be directed to the corresponding author.
